# Modern Sociology

**Published:** 1899-08-26

**Authors:** 


### Modern Sociology.

### THE ETHICS OF SUICIDE.

By Francis Scougall.

One of the most noticeable features of this *fin de siècle* is certainly the remarkable increase in the number of suicides which are perpetually recorded with very little comment in the daily papers. Were a file of newspapers dating a hundred years ago to be examined they would be found to contain only very rare instances of release from temporary troubles having been sought by means of "shuffling off the mortal coil" altogether. Our forefathers were, it seems, somewhat more wise in their generation, or perhaps only more phlegmatic; but they certainly were not prone to perpetrate the enormous folly of escaping painful circumstances, which a day or an hour might change, by an irrevocable plunge into the mysterious certainties of the unknown.

The present rage—I can give it no milder name—for self-destruction is not confined to any special class. It prevails among the upper ten thousand as much as among the various ranks of the so-called submerged, although the wide prevalence of society suicides are more discreetly veiled from public notice, under verdicts of heart failure or other undiscernible maladies, than by the broad intimation, which characterises such voluntary exits from life among the lower orders, as the result of temporary insanity. Generally speaking, neither in the higher ranks of life or the lowest are the persons who commit suicide in the least insane. For the present we will leave the aristocratic departures from an unsatisfactory life, to deal with the causes which send ignorant and illiterate persons to a voluntary grave.

Among the upper ranks it is not, at all events, grinding poverty or pressure of physical discomfort which cannot be averted that drives them to escape from an undesirable existence. Among those who have to earn their living it is a main factor in their more immediate impulse to end the misery which presses unendurably upon them for the time being. Cold, hunger, the sight of starving children and despairing wife, the wretchedness of a bare and squalid home, or it may be no home at all but the open streets—these are not unnaturally the causes which, as a final result, send both men and women over the bridge into the deepest water they can find. But these are only the reasons which operate on the surface, as it were, of their intelligent consciousness. There are underlying sources of despair working on the lower types of mind—that are, in fact, the remote influences goading human beings to accomplish a cessation of the vitality which has become mere suffering. The most potent of these is undoubtedly the conviction, firmly welded into the ideas of most of the lower orders by socialistic lectures and atheistical publications, that there is absolutely no survival of life after this short, uncertain existence; that death is the final end of all; that the sentient spirit expires with the body, which moulders into senseless dust; and that there is absolutely no Supreme Judge to take cognisance of the blotting out of a life with which none but the suicide himself has any concern whatever. The manner in which this theory of human existence is now held determinately by the vast majority of our lower populations was graphically stated to the writer by a woman who was one only, out of a very considerable number of both sexes who had preceded her, in announcing their indomitable determination to put an end to their lives so soon as they found them finally unbearable. She had had a career of the utmost infamy, and intended, as she said, to continue it as long as she could make it pay. "Now, look here," she said, "all that talk about a God and a Judge and a judgment has not a word of truth in it—there is nothing of the kind; there is nobody but myself who has anything to do with settling for me, living or dying. We come into the world as the beasts do—the dogs and the cats, and the sheep and the oxen which we use for food, and when we go out of the world again, however it is done, there is an end of us—an end of everything. No more thinking or talking or feeling—all that is just like the flame of a candle that you can blow out and there is nothing to be seen of it any more for ever. Our bodies are all that's left just to be huddled out of sight as quick as possible and left to rot away so that they tell me the ground under our feet is nothing now but dead folk's dust. It is just as if we had never existed. Well, then, since this life is all we have got or shall ever have, there is nothing to be done but to go on with it as long as there is any good or pleasure to be got out of it, but when it turns to deadly dulness, or want, or pain, why on earth should we let it keep its hold on us? Not such fools! Not me or my companions! Out we go by the water, or the poison, or the razor. That's over in a moment, and all's done. We know nothing more about it. That's what I shall do, and that is what every one of my friends and lovers will do. It all rests on money. As long as I can any way get as much of that as I need to enjoy myself with just as I please, I'll go on; when that stops, I stop, too; and good-bye to everything!" Although not always expressed with the coarseness used by this woman, that is practically the creed of the suicide in the slums and crowded quarters of our great cities. If the faintest glimmer of a hope remained to them that there might be a future state more beneficent than their present outward machinery of life, or a shadow of fear that judgment might overtake them hereafter, it would at least, to some extent, influence them against a leap in the dark; but there is meanwhile the consciousness that their own idleness or evil ways or reckless folly have brought them to this pass, seems only to strengthen the desire to annihilate sensation.

There is one very remarkable feature in the suicides of persons in that class of life which does not seem to have received the attention it deserves, either scientifically or medically. There are frequently cases where self-destruction has literally appeared to have been assisted by some exterior force quite apart from the suicide's own action. We can best explain our meaning by giving in illustration a case of which we know all the details. We can briefly cite the instance of a man who from full health arrived at death in about ten minutes by holding his face in a basin which contained only a few inches of water. Medical experts one and all declared that it was to them inconceivable that the purely physical necessity to relieve the agony of suffocation did not absolutely compel him to raise his face for a second from the scanty measure of water, and breathe. Even if he tried again and again to force himself to remain with his breath choked till it ceased, they held that the bodily instinct would for a second at least always overcome him. It is certain, however, that he was entirely alone in the room, where he was speedily found dead.

